# Functional Proteomic Profiling of Triple-Negative Breast Cancer

**DOI:** 10.3390/cells10102768

**Published:** 2021-10-15

**Authors:** Irina Gromova, Jaime A. Espinoza, Morten Grauslund, Eric Santoni-Rugiu, Maj-Lis Møller Talman, Jan van Oostrum, José M. A. Moreira

**Affiliations:** 1 Genome Integrity Unit, Danish Cancer Society Research Center, Cancer Proteomics Group, DK-2100 Copenhagen, Denmark; iig@cancer.dk; 2 SciLifeLab, Department of Medical Biochemistry and Biophysics, Division of Translational Medicine and Chemical Biology, Karolinska Institute, Solna, SE-17176 Stockholm, Sweden; jaime.espinoza.ruiz@gmail.com; 3 Department of Pathology, Diagnostic Center, Copenhagen University Hospital, DK-2100 Copenhagen, Denmark; Morten.Grauslund@regionh.dk (M.G.); eric.santoni.rugiu@regionh.dk (E.S.-R.); maj-lis.moeller.talman@regionh.dk (M.-L.M.T.); 4 Clinical Proteomics Center, Luxembourg Institute of Health, 1445 Strassen, Luxembourg; drjvo2@bluewin.ch; 5 Department of Drug Design and Pharmacology, University of Copenhagen, DK-2100 Copenhagen, Denmark

**Keywords:** breast cancer, triple negative breast cancer, proteomics, signaling pathway profiling, reverse phase protein array

## Abstract

Triple-negative breast cancer (TNBC) is a subtype of breast cancer that comprises various disease entities, all of which share a set of common features: a lack of expression of the estrogen receptor, progesterone receptor, and human epidermal growth factor receptor 2, respectively. Because of their receptor status, conventional chemotherapy remains the main therapeutic option for TNBC patients. We employed a reverse phase protein array approach (RPPA), complemented by immunohistochemistry, to quantitatively profile the activation state of 84 actionable key signaling intermediates and phosphoproteins in a set of 44 TNBC samples. We performed supervised and unsupervised approaches to proteomic data analysis to identify groups of samples sharing common characteristics that could be amenable to existing therapies. We found the heterogenous activation of multiple pathways, with PI3 K/AKT/mTOR signaling being the most common event. Some specific individualized therapeutic possibilities include the expression of oncogenic *KIT* in association with cytokeratin 15 and Erk1/2 positive tumors, both of which may have clinical value.

## 1. Introduction

Over the last few decades, we have witnessed substantial improvements in breast cancer (BC) mortality rates [1,2]; downward BC mortality trends are seen in most high-income countries and are essentially a reflection of the improvements that have taken place in the clinical management of BC, together with developments enabling early diagnosis of the disease. Improvements in the clinical management of BC are mainly due to the establishment of more effective combination chemotherapy regimens, the implementation of endocrine therapies in the adjuvant setting, as well as the introduction of novel targeted drugs. Nonetheless, BC is still the leading cause of death from cancer among women, and the future burden of the disease is estimated to increase significantly due to demographic patterns [3]. In the US alone, the number of new BC cases is forecasted to surge from 205,606 patients in the year 2000 to 379,052 by the year 2050 [4]. Worldwide, the number of incident BC cases is expected to exceed three million by 2040 [5]. As these two trends diverge (incidence increasing and mortality decreasing), they have, so far, essentially offset each other. However, as we approach the upper limit of efficacy that the current therapies can achieve, we will need to implement much more effective therapies and/or regimens to be able to maintain the current downward mortality trends.

As mentioned, the therapeutic options currently at the disposal of BC oncologists have undeniably resulted in survival benefits for patients bearing tumors of the estrogen receptor (ER)-positive and human epidermal growth factor receptor 2 (Her2)-positive subtypes, respectively. However, about 12–20% of all breast cancers are negative for ER, progesterone receptor (PgR), and Her2, comprising the triple negative breast cancer (TNBC) group [6,7]. This is a highly heterogeneous disease not only at the molecular level but also at the pathologic and clinical levels.

TNBC is associated with a significantly higher probability of relapse and poorer overall survival in the first few years after diagnosis when compared with other breast cancer subtypes. For this cancer subtype, conventional chemotherapy remains the main therapeutic option as the major currently available BC therapies are based on our ability to target ER and Her2. The limited treatment options, in combination with the more aggressive nature and substantial heterogeneity of these tumors, makes the identification of novel therapeutic targets for this group of patients of particular importance [8,9,10]. Previously proposed classifications of TNBC, both at the transcriptomics level [11,12,13] and the proteomic level [14], identified multiple TNBC molecular subtypes potentially amenable to different treatment strategies. However, the different classifications were not concordant, especially across different modalities, suggesting that some molecular subtypes are not consistent, and underscoring the need for multiple studies across technological platforms and patient cohorts.

We have previously reported a systematic gel-based proteomic profiling study of a prospective cohort of 78 TNBC patients, which resulted in the establishment of a cumulative TNBC protein database containing more than 400 unique entries. An analysis of this database identified a number of proteins as being overexpressed in TNBC samples, which could constitute potential therapeutic targets [15,16]. However, as many of the proteins we identified in this manner did not have a corresponding drug available for clinical use, we undertook a complementary approach. We performed functional proteomic profiling of our TNBC cohort with reverse phase protein arrays (RPPA) to profile pathway activation across samples using a panel of activation-state-specific antibodies. Most of the targeted drugs currently available are directed against signaling pathway intermediates and are designed to interfere with their function. Accordingly, a systematic pathway activation profiling of TNBC samples could potentially identify alternative subtypes of TNBCs that are amenable to a particular therapy, which would be of clinical value. We present here our profiling analysis of the activation state of key signaling mediators, such as Akt or Erk, as well as BC-relevant markers, such as p53, BRCA1, and proliferation markers in TNBC samples.

## 2. Materials and Methods

### 2.1. Sample Collection and Preparation

Fresh snap-frozen tissue biopsies from non-selected retrospective TNBC cases were collected by the Department of Pathology at the Copenhagen University Hospital [15]. Fresh breast tissue samples were flash-frozen in liquid nitrogen immediately following surgery and stored at −80 °C until the time of analysis; on average, no more than 15 min elapsed from tissue excision to freezing. None of the patients had previously undergone surgery involving the breast, and they did not receive preoperative treatment. The patients presented a unifocal tumor with an estimated size >20 mm. The project was approved (KF 01–069/03) by the Copenhagen and Frederiksberg regional division of the Danish National Committee on Biomedical Research Ethics. Written informed consent was obtained from each patient included in the study. Clinicopathological information was provided by the Department of Pathology, Copenhagen University Hospital.

### 2.2. Reverse Phase Protein Arrays (RPPA)

The tissue samples were prepared by solubilizing 20–40 (depending on tumor cell content and area of section) 8 µm-thick cryosections of snap-frozen tissue biopsies in 100 µL ZeptoMARK lysis buffer CLB1 to yield lysates with similar protein concentrations (approximately 2 mg/mL) [17]. Actual protein concentrations were determined using a modified Bradford assay, compatible with the ZeptoMARK lysis buffer CLB1 (Zeptosens, Bayer Technology Services, Witterswil, Germany). All but three samples were spotted at equal total protein concentrations starting from 1.5 mg/mL, adjusted with the ZeptoMARK lysis buffer CLB1. Three samples, TNBCs #13, #37, and #22 had protein concentrations lower than 1.5 mg/mL and were spotted at the available concentrations. All other samples were diluted 1:10 with ZeptoMARK spotting buffer CSBL1, and subsequently further diluted to obtain a dilution series of 0.15, 0.1125, 0.056, and 0.028 mg/mL, respectively, and finally spotted directly onto ZeptoMARK hydrophobic chips. Array spotting and image acquisition and processing were done as previously described [18]. An additional on-chip protein determination was also made to allow for multiple sample comparisons [18]. Samples were spotted onto arrays in duplicates. Relative intensities for each spot were obtained by plotting net spot intensities against protein concentrations of each of the spotted samples. The eight datapoints for each sample were then fitted using a weighted linear least square fit (STATA, StataCorp, College Station, TX, USA) and the relative intensity interpolated at the median protein concentration. Heatmaps and hierarchical clustering were performed using MeV software [19].

### 2.3. Antibodies

All antibodies used in this study as well as their working dilutions for the different technologies are presented in Table S1. Zeptosens have prevalidated over 300 antibodies for use with their RPPA platform using a standardized screening process [20,21]. These antibodies can be used to simultaneously profile pathway response across multiple samples. For this study, a subset of antibodies was selected to specifically profile cancer-relevant pathways. Pathways covered include RAS-MAPK, Rb/cell-cycle, and PI3 K-Akt signaling axes, as well as DNA repair and apoptosis. A complete list of validated antibodies is available from Bayer Technology Services (www.bayertechnology.com/fileadmin/_migrated/content_uploads/Flyer_BTS_Zeptosens_List_260914.pdf; accessed on 14 February 2020).

### 2.4. Immunohistochemistry (IHC) of Formalin-Fixed, Paraffin-Embedded (FFPE) Samples

Fresh tissue samples were partitioned immediately following surgery, with a part being snap-frozen and and a fraction fixed in neutral buffered formalin and paraffin embedded for IHC-based analyses. IHC analysis were performed on five-μm sections cut from the FFPE blocks essentially as previously described [15].

### 2.5. Quantitative Assessment of IHC Staining

Computer-aided analysis of digitized whole slide images was used for quantitative comparison of immunohistochemical stainings. Slides were scanned using a NanoZoomer-XR Digital slide scanner (Hamamatsu Photonics, Hamamatsu City, Japan). Full section whole slide images were analyzed with QuPath digital pathology image analysis software (QuPath v0.2.3), an open-source imaging software [22]. Two metrics for IHC staining were used: the percentage of staining carcinoma cells (% positive cells), and a composite score, calculated based on the extent and intensity of staining (3× % of strongly staining cells + 2× % of moderately staining cells + 1× % of weakly staining cells, giving a range of 0–300).

### 2.6. DNA Purification and Sequencing

Genomic DNA was collected from four 10 µm-thick FFPE tissue sections of and purified using the QIAamp DNA-mini kit on a Qiacube instrument (Qiagen, Hilden, Germany) according to manufacturer’s instructions. Concentration and purity of isolated DNA were measured on a Nanodrop 2000 instrument. Dideoxynucleotide-sequencing of *c*-*KIT* exons 9, 11, 13, and 17 (standard *c*-*KIT* clinical mutational analysis for prediction of response to TKI therapy; see Table S2) was performed in nested PCR reactions, in which the nest 1 reactions in a volume of 15 µL contained 0.33 µmol/L of each nest 1 primer, 7.5 µL RedEx PCR master mix (Sigma-Aldrich, Brøndby, Denmark), and 50–100 ng genomic DNA. PCR conditions consisted of initial denaturing at 95 °C for 5 min, 25 cycles at 95 °C for 30 s, 55 °C for 30 s, and 72 °C for 30 s, and a final extension at 72 °C for 10 min. The nest 2 reactions were contained in a volume of 50 µL 0.4 µmol/L of each nest 2 primer, 25 µL RedEx PCR master mix (Sigma-Aldrich), and 1 µL of 100-fold diluted nest 1 product. The PCR conditions were the same as for the nest 1 reactions except that 35 cycles were used. PCR products were isolated using the QIAquick PCR purification kit (Qiagen). For sequencing using the T3 and T7 primers, 25 ng purified PCR products were used for BigDye incorporation using the BigDye Terminator 3.1 Cycle Sequencing Kit (Thermo Fisher Scientific, Waltham, MA, USA) and sequenced on an ABI3500 DX DNA sequenator according to the manufacturer’s instructions.

### 2.7. Data Analysis

Data were first normalized by transforming values using the mean and the standard deviation of the row of the matrix to which the value belongs using the following formula: Value = [(Value) − Mean(Row)]/[Standard deviation(Row)]. Samples and genes were then median centered and clustered using Pearson correlation and average linkage. GraphPad Prism (v.9.2.0; GraphPad Software, San Diego, CA, USA) was used for one-way and two-way analyses of variance (ANOVA), as well as Fischer’s exact test and Kaplan–Meier survival analysis. In all cases, an alpha value of 0.05 was used. The knowledge-based platform MetaCore (Thomson Reuters, Spring Garden, PA, USA) was used for pathway analysis.

## 3. Results

### 3.1. RPPA-Mediated Functional Profiling of Tnbcs

In order to identify novel therapeutic options for the clinical management of TNBC, we profiled a cohort of TNBC patients (status defined by a lack of ER, PgR, and HER2 expression in immunohistochemical assays) [15] by an RPPA analysis of a set of 84 signaling pathway intermediates using phospho-specific antibodies (listed in Table S1).

**Figure 1:**
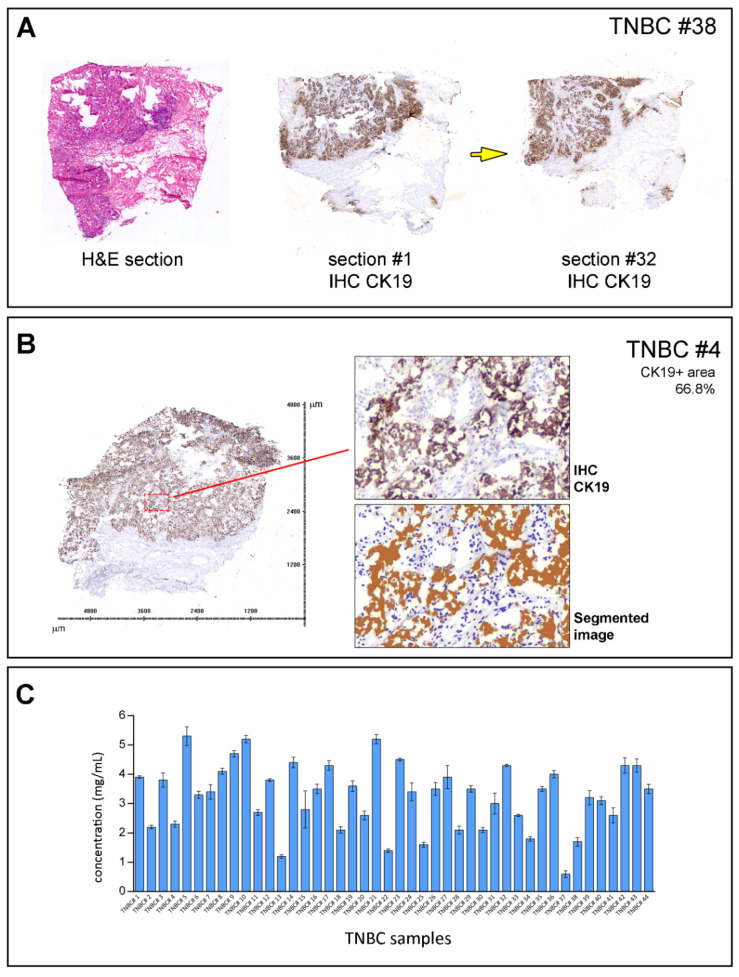
Sample preparation workflow. (**A**) Sample biopsies were serially sectioned and first and last sections stained with hematoxylin and eosin (HE) and cytokeratin 19 (CK19; an epithelial cell marker) for pathology reevaluation. Yellow arrow indicates section order. (**B**) Tandem sections were stained with CK19 to assess the tumor cell area for each sample. Tissue sections stained with CK19 were scanned, and images were segmented and analyzed with image processing software (Visiopharm, Hørsholm, Denmark), which allowed for the quantification of relative tumor cell content. Only samples with tumor cell content >60% were included in this study. (**C**) Lysates were prepared by solubilizing cryosections of snap-frozen tissue biopsies in CLB1 lysis buffer and total protein concentrations determined.

**Figure 2:**
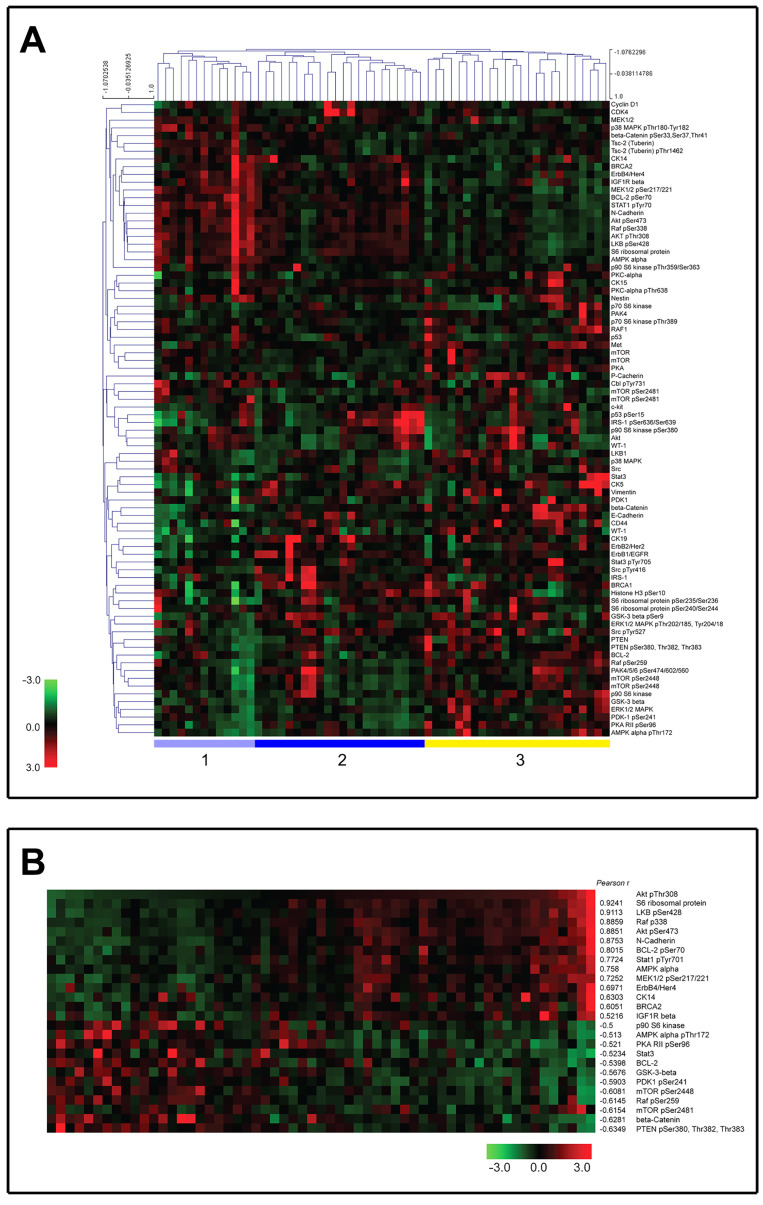
RPPA analysis of 44 TNBC samples. (**A**) Signal intensities were normalized and log2 converted signals were used for an unsupervised hierarchical cluster analysis. A black color indicates the median expression level calculated for a particular protein or phosphorylation across all samples; higher level expression than median is shown as a red color, and a green color refers to a lower than average abundance. Sample names and protein names are listed above and on the right-hand side, respectively. Clusters 1 through 3 are defined at the bottom of heatmap. (**B**) Heatmap of proteins with a Pearson correlation rank above 0.5, sorted by the phosphorylation status of Akt Thr−308.

To minimize the effect of the cellular content of the samples on the results, only samples with >60% tumor cell content, evaluated by CK19 stainings, were included in the study (Figure 1A,B). Hematoxylin- and eosin-stained (H&E) sections of each sample were reviewed by a pathologist to do a coarse determination of the percentage of tumor cells. In addition, we analyzed the tissue sections for CK19-positive cells (CK19+) as this epithelial marker is ubiquitously expressed by mammary epithelial cells. The proportion of cells was only used as an inclusion criterion for the samples but not as a normalization parameter. A total of 44 TNBC tumor samples out of the original 78 samples were found to fulfill the inclusion criterion and were included in this study. Lysates were prepared by solubilizing cryosections of snap-frozen tissue biopsies, and the samples were arrayed onto a hydrophobic chip surface by spotting. An on-chip protein determination was made, allowing for multiple sample comparisons (Figure 1C). The arrayed samples were then probed with phospho-specific and total protein antibodies to measure the activity status of multiple signaling pathways or networks [21]. Antibody microarray data, consisting of relative intensity values interpolated at the median protein concentration for each sample (see Section 2), were analyzed by two-way unsupervised hierarchical clustering, as described [23]. The unsupervised hierarchical clustering analysis of proteomic data identified three major clusters (clusters one through three, Figure 2A). These clusters were characterized by well-known signaling modules, such as PI3 K/Akt in cluster one. Cluster two was characterized by samples with high expression levels and the phosphorylation of most of the proteins involved in the Akt-mTOR pathway. Interestingly, cluster two presented high levels of Akt expression and phosphorylation but lower levels of activation of downstream effectors compared to cluster one. Cluster two also presented low levels of PTEN and higher levels of phospho-IRS1, an adaptor protein in charge of conveying signaling from the IGF-1 receptor to activate the PI3 K/Akt signaling pathway. The remaining cluster showed highly heterogeneous pathway activation (Cluster three). Overall, the analysis confirmed the extensive molecular heterogeneity of TNBCs and the prevalence of the PI3 K/AKT/mTOR signaling axis in these cancers.

### 3.2. Pathway-Restricted Analysis of RPPA Data

We then performed a pathway-restricted analysis of our data, limiting the analysis to proteins known to be within the Akt pathway (Figure S1). Figure 2B shows data clustered by the phosphorylation state of Akt. We performed a Pearson correlation analysis between the phosphorylation levels of all of the analytes and Akt phospho-Thr308 for all 44 TNBC samples. The proteins with a Pearson correlation of >0.5 and <−0.5 sorted by the expression of Akt phospho-Thr308 are shown in Figure 2B. As one may expect, several members of the Akt signaling pathway itself were on this list, such as S6 ribosomal protein. We also found that the expression of two different non-canonical Akt protein targets, N-cadherin and erbB4, were highly correlated with Akt phosphorylation (Figure 2B). Phospho PTEN was the protein with the highest anti-correlation with phospho Akt. The activation of Akt involves the phosphorylation of two residues, threonine 308 (Thr308) and serine 473 (Ser473). Both Akt Thr308 and Ser473 phosphorylations were highly correlated (r = 0.8851; *p* value < 0.0001) among the 44 TNBC samples (Figure 2B), suggesting that both phosphorylation states were co-regulated among TNBCs tissues.

### 3.3. Cytokeratin 15 Expression in Tumor Cells Was Associated with Erk1/2 Signaling

**Figure 3:**
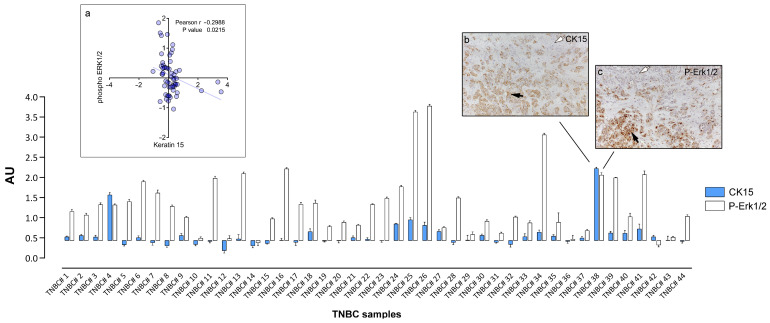
Expression of CK15 and Erk1/2 phosphorylation in TNBC samples determined by IHC. Normalized RPPA values for CK15 and P-Erk1/2 were plotted using as baseline value the threshold RFI value of samples found negative by IHC analysis (subpanel **a**). IHC images of a heterogenous sample containing cancer cells positive (black arrow) and negative (white arrow) for CK15 (subpanel **b**) or P-Erk1/2 (subpanel **c**), respectively are presented for illustration purposes. Magnification, 20×.

Although TNBCs are distinct from the basal-like intrinsic molecular subtype of breast carcinomas, they show a substantial overlap with these tumors [11,24]. Basal-like breast cancers are characterized by the expression of basal keratins and have previously been suggested to contain a high proportion of stem-cell-like cells [25]. We and others have identified cytokeratin 15 (CK15) as a marker of mammary progenitor or stem cells [26,27,28,29], and a recent study reported a 15-gene mammary stem cell signature that comprised CK15 and showed prognostic value in TNBCs [27]. We had included CK15 in our RPPA analysis to ascertain whether CK15 expression was associated with a particular signaling event in TNBCs that could be targetable. A correlation analysis of our RPPA data showed that the expression of CK15 was associated with the expression of v-kit Hardy-Zuckerman 4 feline sarcoma viral oncogene homolog (c-Kit) protein and Erk1/2 phosphorylation (inset a; Figure 3A). The c-Kit association was consistent with our previous report that the expression of c-Kit correlates with that of CK15 in ER-negative breast epithelial cells [30].

The levels of expression of CK15 and Erk1/2 phoshorylation in tumor cells were verified by a quantitative IHC analysis of the interleaved tissue sections to those sections used to prepare lysates for the RPPA analysis (Figure 3). The stained images were scored by computer-aided analysis (see Section 2). The scores were normalized, and the median was centered and plotted (Figure 3). We found that the samples that displayed a strong signal in our RPPA analysis did correspondingly in the IHC. Conversely, samples with a low or no RPPA signal showed no immunoreactivity in the IHC. The samples with the heterogenous expression of CK15, exhibiting CK15-negative tumor regions (Figure 3, inset b, white arrow) in the vicinity of cells with marked CK15 expression (Figure 3, inset b, black arrow), showed a similar pattern of staining of P-Erk1/2 (Figure 3, inset c, white and black arrows, respectively). The IHC analysis confirmed that CK15 expression was associated with c-Kit protein expression (*p* < 0.001; two-way ANOVA multiple comparison analysis) and Erk1/2 phosphorylation (*p* < 0.001; two-way ANOVA multiple comparison analysis).

### 3.4. Expression of Oncogenic C-Kit Defines a Subset of Tnbcs

**Figure 4:**
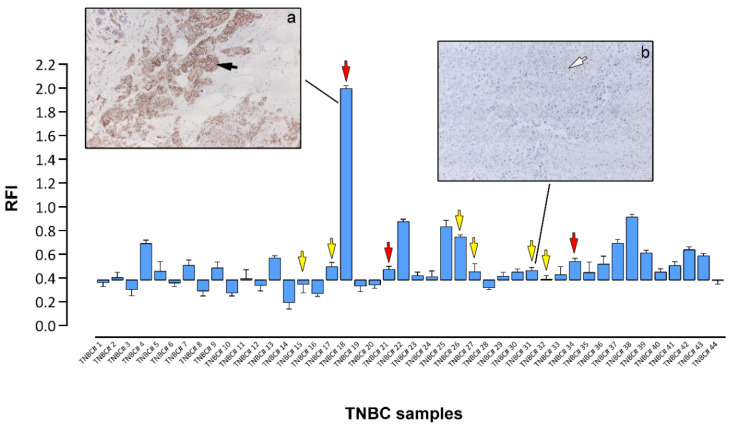
Expression of c-Kit in TNBC samples. Normalized RPPA values for c-Kit were plotted using as baseline value the relative fluorescence intensity (RFI) values of samples found negative by IHC analysis. IHC images of samples stained for c-Kit and showing strong (black arrow, subpanel **a**) or no expression (white arrow, subpanel **b**) of c-Kit are presented for illustration purposes. Sequenced samples are indicated by arrows (yellow arrows no mutations detected, red arrows oncogenic mutations present). Magnification, 20×.

One of the ongoing research activities in our lab addresses the need to define the molecular phenotypes of the various cell types and precursors generated by the stem cell hierarchy in the breast epithelium. To that effect, we have immunophenotyped normal human luminal epithelial cells and identified CK15 and c-Kit as protein biomarkers of normal undifferentiated luminal cells in resting acini [30]. C-Kit is a tyrosine kinase receptor that mediates the activation of several effector-signaling pathways involved in key cellular functions, such as cell survival, proliferation, and differentiation [31]. Since c-Kit was previously reported to be overexpressed in approximately 25% of samples in a large TNBC cohort [32], we had included c-Kit in our RPPA profiling analysis. We found that a subset of our samples expressed c-Kit at substantially high levels (Figure 4). As before, we could validate these results with an IHC analysis, showing that samples with a strong RPPA signal for c-Kit presented moderate to strong immunoreactivity (Figure 4, panel a, black arrow), whereas samples with a low RPPA signal had no detectable immunoreactivity for c-Kit (Figure 4, panel b, white arrow). Under the conditions of the IHC assay, 9 out of the 44 TNBC samples (20%) expressed c-Kit.

Oncogenic c-Kit receptor tyrosine kinase “driver” mutations have been identified in a number of cancers, such as acute myeloid leukemia (AML), melanoma, in mast cell leukemia, and in gastrointestinal stromal tumors (GIST) [33], and tyrosine kinase inhibitors (TKIs), such as imatinib or sunitinib, with activity against c-Kit routinely used in the treatment of these cancers. *KIT* mutation testing is important for the prediction of the response to TKI therapy. Commonly, mutations map to the extracellular region (exon 9), juxtamembrane region (exon 11), and kinase domain (exons 13, and 17). We performed a mutational analysis of *KIT* in samples (Figure 4, yellow and red arrows) for which we had biological material available to analyze (Table 1). We found somatic mutations in three of the cases (Figure 4, red arrows). In one of these (Figure 4, #18), c-Kit was expressed at very high levels, suggesting that the pharmacological inhibition of c-Kit in this case could have been of use in the management of the disease.

## 4. Discussion

### 4.1. Profiling of Pathway Activation

Novel therapies for triple negative breast cancer (TNBC) are necessary to improve the dismal prognosis of this disease. The purpose of this study was to identify molecular subtypes of TNBC based on actionable signaling intermediates, thus defining possible therapies or therapy combinations that could be of clinical value in the management of TNBC patients. We employed an RPPA approach to quantitatively profile the activation state of 84 cancer-related key signaling intermediates and phosphoproteins in a set of 44 TNBC samples. We tried to identify groups of samples sharing common characteristics that may be amenable to existent therapeutic approaches with the aim of developing more effective therapeutic regimens for TNBCs. The results from the RPPA analysis can be easily adapted to a clinical setting through the development of immunohistochemical assays. We could identify three major clusters, where the first cluster (Figure 2A, cluster one) showed PI3 K/Akt signaling, the second cluster was defined by Akt-mTOR pathway activation but with lower levels of activation of canonical Akt downstream effectors, and the third cluster was characterized by the heterogenous activation of multiple pathways. 

As a part of The Cancer Genome Atlas (TCGA) Project, protein expression data have been generated over a large number of breast tumors using RPPAs [34]. This publicly available collection of cancer functional proteomics data includes a large set of basal-type tumors (n = 118) that have been profiled for 245 protein markers. Although only 52 out of 84 markers were identical to those in our own study, we investigated whether we could reiterate our observations. The supervised hierarchical clustering of these 52 markers largely confirmed our analysis (Figure S2A). As with our own dataset, we could find the clusters defined by specific signaling modules, the most prominent of which was PI3 K/Akt signaling (Figure 2A and Figure S2).

Among the 25 proteins we found to be statistically correlated with the phosphorylation of Akt at Thr−308, 9 had a Pearson rank >0.7 (Figure 2B). The expression of two different non-canonical Akt protein targets, N-cadherin and erbB4, was highly correlated with Akt phosphorylation (Figure 2B). The most straightforward conclusion is that both proteins are, directly or indirectly, under the regulation of Akt. Indeed, there have been previous reports in the literature showing an association between ERBB4 expression and Akt phosphorylation in TNBCs [35], as well as between N-cadherin expression and Akt signaling during epithelial mesenchymal transition (EMT) [36]. However, a direct causal relationship cannot be excluded either as Rieger-Christ and colleagues showed that an increase in the phosphorylation of Akt at serine−473 was detected in N-cadherin transfectants, suggestive of N-cadherin-mediated Akt activation in bladder cancer cell invasion [37].

We also found that Akt phosphorylation at Thr308 and Ser473 were highly correlated (r = 0.8851) among the 44 TNBC samples (Figure 2B), suggesting that both phosphorylation states were co-regulated among TNBCs tissues. Although the phosphorylation of Thr308 is sufficient to activate some downstream effectors, the additional phosphorylation of Ser473 enables the full activation of Akt [38]. A similar trend was also observed in the analysis of the phosphorylation status of the TCGA breast cancer study [39]. Stemke-Hale and colleagues showed that Akt phosphorylation was at significantly higher levels in PTEN-low compared with PTEN-high breast cancers [40].

Recent research into the biology of TNBCs has resulted in the identification of several possible novel targets for pathway-driven therapeutics in this disease [41,42]. As one may expect, major signaling modules involved in cell survival and proliferation, such as mitogen-activated protein kinase (MAPK) and PI3 K/Akt signaling, are among the prominent candidate target pathways in TNBCs, with several clinical trials already underway. Our analyses used phospho-specific antibodies to measure the phosphorylation at the Ser473 and Thr308 residues of Akt, using these as activation readouts for PI3 K/Akt signaling. This was also the case for two of the three major subfamilies of the MAPK family for which we had reliable reagents: the extracellular signal-regulated kinases (ERKs) (Thr202/185, Tyr204/187), and the p38 kinases (Thr180/Tyr182). The levels of activation for these specific pathways in our TNBC samples can be seen in Figure 2. Only in a few cases were we unable to detect the activation of any of these three pathways (Figure 2, samples #12 or #14), but, in most cases, at least one of these signaling pathways was highly active (for example, sample #34 for Erk1/2, or sample #41 for p38), suggesting that most TNBCs would be amenable to pathway-directed therapies that target MAPKs and/or Akt. However, one additional aspect also needs to be considered: TNBC are heterogeneous in terms of genetic alterations of the PI3 K/Akt pathway, where 7–23% of cases have a mutation in the PIK3 CA gene with a high degree of mutual exclusivity with PTEN mutation/loss (11–35%) and INPP4 B loss (30%), whereas the latter two genes have a major degree of overlap between them [39,43]. In addition, many components of the RAS-RAF-MEK pathway are amplified in TNBC, including KRAS, BRAF, and EGFR, among others [39]. This implies that genetic alterations of the PI3 K/Akt pathway (and other interconnected pathways) can partly explain the heterogeneity observed at the level of activation/inhibition of downstream effectors.

One last point to be taken into consideration is that only samples with >60% tumor cell content, evaluated by CK19 stainings, were included in the study. This approach was taken to ameliorate the technical difficulties associated with samples consisting of a mixture of tumor cells and normal cells, which is especially challenging when the tumor cell content is very low. Nonetheless, the RPPA data we generated from the tissue lysates result from a blend of diverse cell populations (e.g., endothelial cells, stromal cells, normal epithelial cells, tumor cells, and immune cells), thus producing an averaging effect. With the exception of the specific markers investigated by IHC, we cannot easily determine the cell of origin of a given signaling intermediate in our RPPA data.

### 4.2. CK15, c-Kit, and Erk1/2 Signaling

We could also further identify some specific individualized therapeutic possibilities based on our RPPA profiling, such as the expression of c-Kit or CK15 in Erk1/2 positive tumors. Among the 44 TNBC samples examined, two were strongly positive for CK15 (Figure 3; samples #4 and #38), and both showed high-levels of Erk1/2 phosphorylation (phospho-Thr202/185, Tyr204/187). This association was a non-reciprocal relation as samples with high levels of phospho-Erk1/2 did not necessarily express CK15 (Figure 3; samples #11 or #16), suggesting the possibility that Erk1/2 pathway activation may be driving CK15-positive cells in TNBCs, identifying the Erk MAPK signaling pathway as a potential drug target for CK15-positive TNBC tumors. The Erk signaling axis has been previously shown to regulate the expansion and tumorigenicity of breast cancer stem-like cells [44,45].

In relation to c-Kit, we found that 9 out of the 44 TNBC samples had strong expressions of c-Kit, which was confirmed by IHC (Figure 4). This frequency of c-Kit expression (20.4 percent of cases) was in line with previous reports [32,46]. This prevalence was also confirmed by the TCGA data, with c-Kit expression defining a subgroup of patients (Figure S2). In addition, we found that one of the TNBC samples with strong c-Kit protein expression had an oncogenic mutation in *KIT* (Table 1). Although c-kit expression is common in TNBCs, reports on the presence of c-kit mutations are scarce [47]. In conclusion, we found that high-level c-Kit expression is a frequent event in TNBCs, and activating mutations were also present, which makes the case for further research into the potential effect of c-Kit inhibitors on TNBCs in the presence of activating mutations in a clinical trial setting.

## Figures and Tables

**Table 1:** Mutational Analysis of *KIT*.

Patient	*c*-*KIT*			
	Exon 9	Exon 11	Exon 13	Exon 17
TNBC #15	wild-type	wild-type	wild-type	c.2394 G > T (silent/polymorphism)
TNBC #17	wild-type	wild-type	wild-type	wild-type
TNBC #18	c.1414 G > A (silent/polymorphism)	c.1678_1680 del3 p.Val560 del	wild-type	wild-type
TNBC #21	wild-type	c.1660_1662 del3 p.Glu1554 del	wild-type	wild-type
TNBC #26	wild-type	wild-type	wild-type	wild-type
TNBC #27	wild-type	wild-type	wild-type	wild-type
TNBC #31	wild-type	wild-type	wild-type	wild-type
TNBC #32	wild-type	wild-type	wild-type	wild-type
TNBC #34	wild-type	c.1678_1680 del3 p.Val560 del	wild-type	wild-type

## Data Availability

The data presented in this study are available on request from the corresponding author. The data are not publicly available due to privacy law restrictions.

## Supplementary Materials

The following are available online at https://www.mdpi.com/article/10.3390/cells10102768/s1, Supplementary Figure S1: MetaCore analysis of RPPA data showed that several proteins within the Akt signaling pathway were affected in the examined samples in signaling modules related to (A) cell migration and survival, but also (B) in connection to other pathways. Supplementary Figure S2: RPPA data for TNCB breast cancer Columns are centered; unit variance scaling is applied to columns. Rows are clustered using Euclidean distance and average linkage. Columns are clustered using correlation distance and average linkage. Supplementary Table S1. List of all antibodies used in this study. Supplementary Table S2. Primer sequences for sequencing of c-KIT exons 9, 11, 13 and 17.

## Abbreviations

IHC: immunohistochemistry; 2 D PAGE, two-dimensional polyacrylamide gel electrophoresis; Her2, human epidermal growth factor receptor 2; ER, estrogen receptor; PgR, progesterone receptor; RPPA, Reverse Phase Protein Array.
