# Supplementary material for: Functional Proteomic Profiling of Triple-Negative Breast Cancer
Source: Cells. 2021 Oct 15;10(10):2768. doi: 10.3390/cells10102768 (PMC8535076; doi:10.3390/cells10102768)

Supplementary Table S1. List of all antibodies used in this study

Antigen	*Supplier*	*Cat. Nr.*	*Dilution (fold)*	
			RPPA	*IHC*
Akt	Invitrogen (Biosource)	44-609G	250	100
Akt (phospho-Thr308)	Cell Signaling Technologies	9275	500	80
Akt (phospho-Ser473)	Invitrogen (Biosource)	44-621G	500	80
AMPK α	Cell Signaling Technologies	2532	250	-
AMPK α (phospho-Thr172)	Cell Signaling Technologies	2535	500	-
Bcl-2	Epitomics	1017-1	500	-
Bcl-2 (phospho-Ser70)	Cell Signaling Technologies	2827	250	-
β-Catenin	Cell Signaling Technologies	9562	500	-
β-Catenin (phospho-Ser33,Ser37,Thr41)	Cell Signaling Technologies	9561	1000	-
BRCA1	Cell Signaling Technologies	9010	250	-
BRCA2	Cell Signaling Technologies	9012	500	-
Cbl (phospho-Tyr731)	Epitomics	1873-1	1000	-
CD44	Agilent (Dako)	M7082	200	-
CDK4	Cell Signaling Technologies	2906	1000	-
c-Kit	Agilent (Dako)	A4502	1000	250
Cyclin D1	Cell Signaling Technologies	2926	500	-
Cytokeratin 14	Labvision (Neomarkers)	MS-115	200	-
Cytokeratin 15	AVIVA Systems Biology	AVARP00005_P050	2000	2500
Cytokeratin 19	Labvision (Neomarkers)	MS-198	1000	-
Cytokeratin 5	Epitomics	2290-1	1000	-
E-Cadherin	Cell Signaling Technologies	3195	250	-
ErbB-1/EGFR	Cell Signaling Technologies	2232	1000	-
ErbB-2/Her-2	Agilent (Dako)	A0485	500	-
ErbB-4/Her-4	Cell Signaling Technologies	4795	500	-
GSK-3-β	Cell Signaling Technologies	9315	500	-
GSK-3-β (phospho-Ser9)	Cell Signaling Technologies	9322	500	-
Histone H3 (phospho-Ser10)	Millipore (Upstate)	06-570	500	-
IGF-1R-β	Cell Signaling Technologies	3027	500	-
IRS-1	Cell Signaling Technologies	2382	1000	-
IRS-1 (phospho-Ser636,Ser639)	Cell Signaling Technologies	2388	1000	-
Ki67	Becton Dickinson	610968	500	-
LKB (phospho-Ser428)	Cell Signaling Technologies	3482	250	-
LKB1	Cell Signaling Technologies	3047	500	-
MEK1/2	Cell Signaling Technologies	9122	1000	-
MEK1/2 (phospho-Ser217/221)	Cell Signaling Technologies	9154	1000	-
c-Met	Cell Signaling Technologies	4560	500	-
c-Met (phospho-Tyr1234)	Signalway	11227-1	1000	-
mTOR	Cell Signaling Technologies	2972	250	80
mTOR (phospho-Ser2448)	Cell Signaling Technologies	2971	250	80

mTOR (phospho-Ser2481)	Millipore (Upstate)	09-343SP	1000	200
N-Cadherin	Cell Signaling Technologies	4061	250	
Nestin	Santa Cruz Biotechnology	sc-23927	100	
p38 MAPK	Cell Signaling Technologies	9212	500	100
p38 MAPK (phospho-Thr180,Tyr182)	Cell Signaling Technologies	4631	1000	150
p44/42 MAPK	Cell Signaling Technologies	9102	500	100
p44/42 MAPK (phospho-Thr202/185,Tyr204/187)	Cell Signaling Technologies	9101	2000	250
p53	Cell Signaling Technologies	9282	1000	-
p53 (phospho-Ser15)	Cell Signaling Technologies	9284	500	-
p70 S6 Kinase	Cell Signaling Technologies	9202	250	-
p70 S6 Kinase (phospho-Thr389)	Epitomics	1175-1	500	-
p90 S6 Kinase (Rsk1-3)	Santa Cruz Biotechnology	sc-231	5000	-
p90 S6 Kinase (Rsk1-3) (phospho-Ser380)	Cell Signaling Technologies	9341	250	-
p90 S6 Kinase (Rsk1-3) (phospho-Thr359,Ser363)	Epitomics	2006-1	500	-
PAK4	Cell Signaling Technologies	3242	500	-
PAK4/PAK5/PAK6 (phospho-Ser474/602/560)	Cell Signaling Technologies	3241	250	-
PARP (cleaved Asp214)	Cell Signaling Technologies	9541	500	-
P-Cadherin	Cell Signaling Technologies	2189	250	-
PDK-1	Cell Signaling Technologies	3062	500	-
PDK-1 (phospho-Ser241)	Cell Signaling Technologies	3061	250	-
PKA	Cell Signaling Technologies		1000	-
PKA RII (phospho-Ser96)	Abcam	ab26322	1000	-
PKC-α	Millipore (Upstate)	05-154	500	-
PKC-α (phospho-Thr638)	Abcam	ab32502	1000	-
PTEN	Cell Signaling Technologies	9552	500	-
PTEN (phospho-Ser380,Thr382,Thr383)	Cell Signaling Technologies	9554	250	-
Raf (phospho-Ser259)	Cell Signaling Technologies	9421	500	-
Raf (phospho-Ser338)	Cell Signaling Technologies	9427	500	-
Raf1	Santa Cruz Biotechnology	sc-133	1000	-
Ras (pan)	Becton Dickinson	610001	100	-
S6 Ribosomal Protein	Cell Signaling Technologies	2217	250	-
S6 Ribosomal Protein (phospho-Ser235,Ser236)	Cell Signaling Technologies	2211	1000	-
S6 Ribosomal Protein (phospho-Ser240,Ser244)	Cell Signaling Technologies	2215	250	-
SAPK/JNK	Cell Signaling Technologies	9258	500	-
Src	Cell Signaling Technologies	2109	500	-
Src (family) (phospho-Tyr416)	Cell Signaling Technologies	2101	500	-
Src (phoshp-Tyr527)	Cell Signaling Technologies	2105	500	-
Stat1 (phospho-Tyr701)	Cell Signaling Technologies	9171	500	-
Stat3	Cell Signaling Technologies	9132	500	-
Stat3 (phospho-Tyr705)	Cell Signaling Technologies	9131	1000	-
Tsc-2 (Tuberin)	Cell Signaling Technologies	3612	1000	-
Tsc-2 (Tuberin) (phospho-Thr1462)	Cell Signaling Technologies	3617	500	-
Vimentin	Agilent Technologies (Dako)	M0725	500	-

WT1	Agilent Technologies (Dako)	M3561	100	-
WT1	Santa Cruz Biotechnology	sc-192	250	-

Supplementary Table S2. Primer sequences for sequencing of *c-KIT* exons 9, 11, 13 and 17

Exon	*Primer*	*Primer sequence*	*Fragment (bp)*
9	F-N1	5´- TCTGTTGATTATGAACCTCTAACTTTG	398
	R-N1	5´- CATGGTCAATGTTGGAATGAA	
	F-N2*	5´- AGTATGCCACATCCCAAGTG	333
	R-N2*	5´- TGACTGATATGGTAGACAGAGCC	
11	F-N1	5´- GGCATGATGTGCATTATTGTG	411
	R-N1	5´- TGGCAAACCTATCAAAAGGG	
	F-N2*	5´- TGTTCTCTCTCCAGAGTGCTCTAA	291
	R-N2*	5´- AAACAAAGGAAGCCACTGGA	
13	F-N1	5´- TGAAACTGCACAAATGGTCC	469
	R-N1	5´- AAGCAGTTTATAATCTAGCATTGCC	
	F-N2*	5´- CTTTCGGGAAGGTTGTTGAG	357
	R-N2*	5´- AGCATTGCCAAAATCATATTAAA	
17	F-N1	5´- TGTGAACATCATTCAAGGCG	417
	R-N1	5´- AAATGTGTGATATCCCTAGACAGG	
	F-N2*	5´- TTGATTTTTATTTTTGGTGTACTGAA	352
	R-N2*	5´- TCACAGGAAACAATTTTTATCGAA	

**The N2 nested primers contain T3 and T7 promoter sequences on F-N2 and R-N2, respectively.*

Supplementary Figure S1. MetaCore analysis of RPPA data showed that several proteins within the Akt signaling pathway were affected in the examined samples in signaling modules related to **(A)** cell migration and survival, but also **(B)** in connection to other pathways.

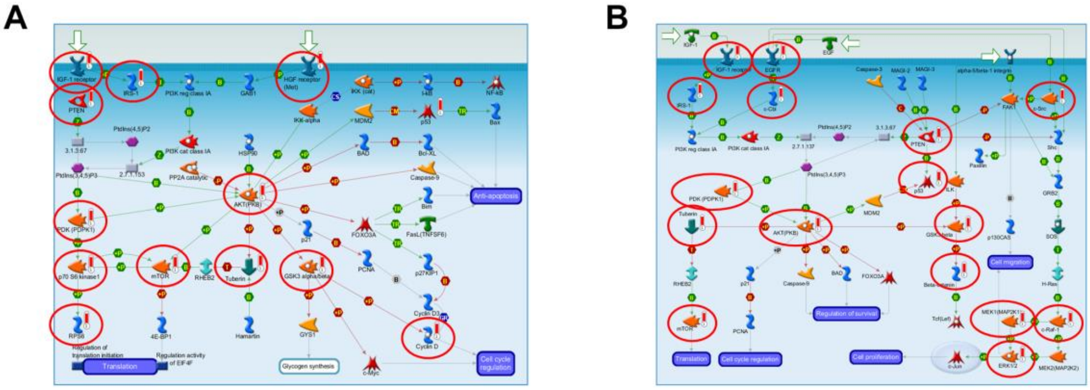

Supplementary Figure S2. RPPA data for TNCB breast cancer Columns are centered; unit variance scaling is applied to columns. Rows are clustered using Euclidean distance and average linkage. Columns are clustered using correlation distance and average linkage.

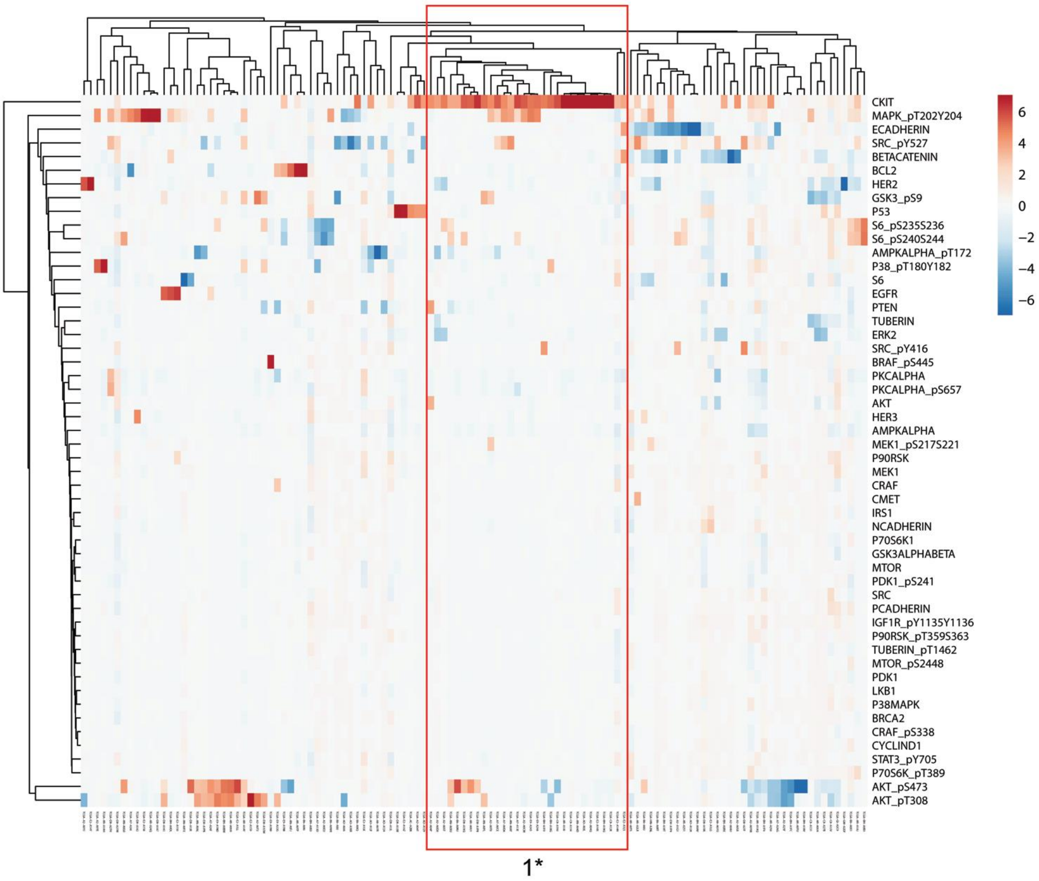

Supplement: Supplementary file 1 [file cells-10-02768-s001.zip › cells-1366021-supplementary.pdf]
